# Review of Speech Outcomes in Cochlear Implant Recipients at a Nascent Cochlear Implant Program

**DOI:** 10.7759/cureus.22543

**Published:** 2022-02-23

**Authors:** Nathan Aminpour, Laura Levin, Mary Finkbone, Michael Morikawa, Melissa Blumgart, H. Jeffrey Kim, Michael Hoa

**Affiliations:** 1 Department of Otolaryngology, Georgetown University School of Medicine, Washington DC, USA; 2 Department of Otolaryngology, MedStar Georgetown University Hospital, Washington DC, USA

**Keywords:** sensorineural hearing loss, speech outcomes, azbio, cnc, cochlear implants

## Abstract

Introduction: The use of cochlear implantation to rehabilitate moderate to profound sensorineural hearing loss has become more widespread; however, the adult utilization rate of cochlear implant candidates is still very less. The study aims to examine the percentage of adult patients in a heterogeneous group of cochlear implant recipients at a nascent cochlear implant program who demonstrate improvements in speech outcomes.

Methods: Speech outcome scores were assessed preoperatively and postoperatively at three, six, and 12-month intervals using consonant-nucleus-consonant (CNC) words and AzBio sentences in quiet. Mean speech outcome scores at each time point and binomial distribution tables with 95% CI were used to assess individual improvement in speech understanding.

Results: 45 patients underwent a total of 49 cochlear implantation surgeries. The mean age at surgery was 62 years. The mean preoperative CNC score in the ear to be implanted was 18%±18, while the mean postoperative CNC score at three, six, and 12 months was 35%±21, 44%±23, and 45%±25, respectively. The mean preoperative AzBio score in the ear to be implanted was 22%±26 while the mean postoperative AzBio score at three, six, and 12 months was 50%±29, 56%±27, and 63%±26, respectively. Of the implantations, 74% (32 of 43) and 69% (22 of 32) showed significant improvement at six months or one year using AzBio and CNC binomial distribution tables, respectively.

Conclusions: Findings demonstrate significant improvements in speech perception following cochlear implantation for patients not benefiting from hearing aid aural rehabilitation. The study provides realistic expectations for new and emerging programs hoping to demonstrate cochlear implant utility for improving patients’ speech outcomes.

## Introduction

The use of cochlear implantation to rehabilitate moderate to profound sensorineural hearing loss has become more widespread. According to the United States (US) Food and Drug Administration (FDA), approximately 324,000 implants were performed worldwide as of 2012 and this number increased to about 600,000 implants in 2016 [1,2]. Despite several studies demonstrating the benefits of cochlear implantation for improving speech understanding [3], sound localization [4], tinnitus management [5], reduced listening effort [6], and improved quality of life [7,8], the adult utilization rate of cochlear implant candidates is still less than 5% [9,10]. Suggested reasons for this underutilization include a lack of awareness, limited access to cochlear implant centers, and non-evidence-based practices [10,11]. However, it is expected that as the FDA expands candidacy [9] and awareness and education about candidacy criteria becomes more widespread, an increasing number of adults will undergo the procedure [12].

Increased utilization of the procedure will require increases in the numbers of cochlear implant surgeons, audiologists, and programs in the US [13]. To appropriately advise patients of speech outcomes resulting from implantation, standardization of expectations across centers is paramount for physicians. Moreover, the literature demonstrates that managing patient expectations for cochlear implantation can contribute to post-implantation follow-up compliance and success [14]. Recent studies have demonstrated significant speech outcome improvement post-implantation in aggregate [9]. Still, a range of outcomes exists [8,15] and the pre-implantation factors that predict outcomes are even less clear [16]. Increased frequency and standardization of post-operative speech testing across the growing implant population may, therefore, provide physicians and audiologists information to better predict patient outcomes.

Our study aims to assess speech understanding outcomes for a heterogeneous group of cochlear implant recipients at a nascent cochlear implant program. Speech performance was assessed preoperatively and postoperatively at three, six, 12, and 24 months using consonant-nucleus-consonant (CNC) words and AzBio sentences. We hope to provide realistic expectations that may demonstrate the utility of the neuroprosthesis in improving patient speech outcomes for patients and guide preoperative and postoperative decisions for physicians.

This article was previously presented as an abstract at the 2021 Combined Otolaryngology Spring Meetings (COSM) on April 10, 2021.

## Materials and methods

Participants

The study was approved by the Institutional Review Board at MedStar Georgetown University Hospital. A total of 75 patients underwent 81 cochlear implantation surgeries from September 2014 to December 2019. All surgeries were performed by either author M.H. or H.J.K. at Medstar Georgetown University Hospital. A retrospective chart review was performed on these 75 patients to collect demographics, preoperative patient histories, intraoperative surgical information, and preoperative and postoperative audiometric testing data at three, six, 12, and 24 months. Of the 75 patients in the initial retrospective chart review, 45 patients (49 surgeries) were included in this study. Exclusion criteria included: non-native English speakers, below the age of 18, pre-lingual bilateral severe to profound sensorineural hearing loss, no preoperative speech outcome scores, lack of compliance with daily device usage defined as greater than or equal to seven hours, single-sided deafness, and hearing loss due to trauma or labyrinthitis ossificans. 

Speech outcome testing

All patients included in this study underwent standardized audiometric evaluation consisting of speech outcome and hearing preservation testing before implantation and at three, six, 12, and 24 months post implantation. Hearing level and cochlear implant performances were measured using CNC monosyllabic word test, AzBio sentence lists in quiet and in noise (+10 dB and +5 dB SNR), and low-frequency pure-tone average (LF PTA) (125, 250, and 500 Hz). AzBio speech testing was performed in the ipsilateral condition. Additionally, all patients were optimally managed with hearing aids in the contralateral ear when possible. Due to a variety of clinical and patient-specific factors (e.g., time constraints, scheduling challenges, cochlear implant equipment issues), not all patients included in this study were evaluated using all audiometric methods across all time points. However, all patients included in this study have preoperative and at least one postoperative speech testing measure of the same type (CNC words or AzBio in quiet). LF PTA, CNC words and AzBio at 24 months, and CNC phonemes and AzBio in noise (+10 dB and +5 dB) across all time points were not used in this study to assess speech outcomes and hearing preservation because of a scarcity of data in the postoperative follow-up period in the patient population. The mean speech performance scores at each time point, mean difference scores between time points, and the significance of these differences as assessed by the binomial distribution tables with 95% CI modeled after Thornton and Raffin [17] were used to assess speech improvement for patients. Patients were also divided into sub-cohorts by type of array (straight or precurved) to assess differences in speech performance.

Statistical analysis 

Statistical analyses were performed with SAS® (SAS Institute Inc., Cary, North Carolina) and JMP® Pro 15 (JMP Statistical Discovery LLC, Cary, North Carolina). Paired t-tests and Wilcoxon signed-rank tests were performed to determine the significance of speech outcomes between time points. Independent sample t-tests and Wilcoxon rank-sum tests were used to compare parametric and non-parametric speech outcome data, respectively, between cohorts based on the type of electrode array. Bivariate analyses using Pearson correlation coefficients were used to determine preoperative variables that correlated with postoperative speech performance. For each speech outcome, we fit a mixed linear model for repeated measures specifying the compound covariance structure to evaluate the main effects of time, gender, age, and electrode type. For all analyses, p-value less than .05 was considered significant.

## Results

Demographics

A total of 45 patients who underwent 49 cochlear implantation surgeries met the inclusion criteria for this study. Of the patients, 49% were female and 39% of implantations were of the left ear. The mean age at surgery for this cohort was 62 years (range 22-91), and the mean duration of deafness measured as age at onset of deafness subtracted from age at surgery was 17 years (range 1-67). Of the 45 patients, three identified as African American, four as Asian, 36 as White non-Hispanic, and two were unknown. Twenty-one of the surgeries used a precurved electrode and 28 of the surgeries used a straight electrode. 

Speech performance 

**Figure 1:**
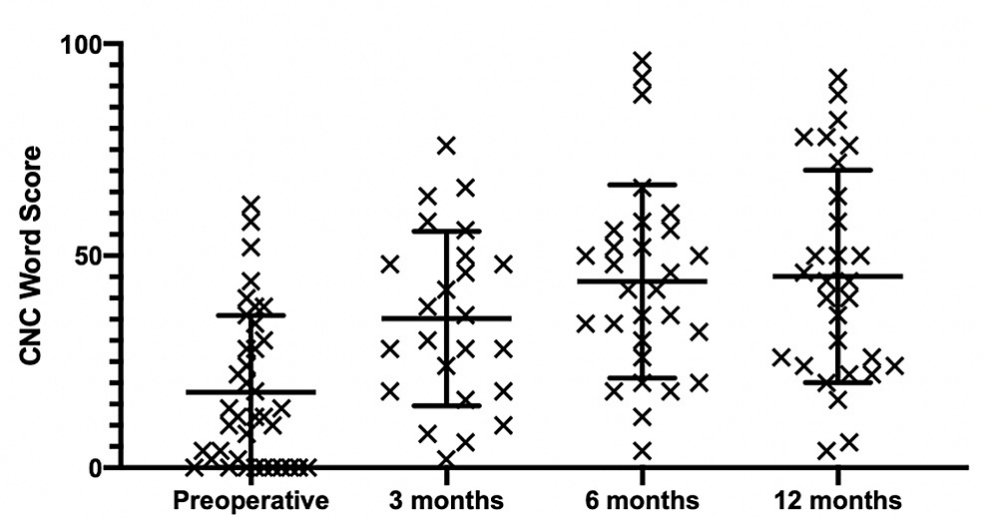
Dot-and-whisker plot of CNC word scores across time points Error bars represent mean and standard deviation. Mean change from preoperative to three months was significant; however, change from three months to six months and six months to 12 months was not significant. CNC: consonant-nucleus-consonant

**Figure 2:**
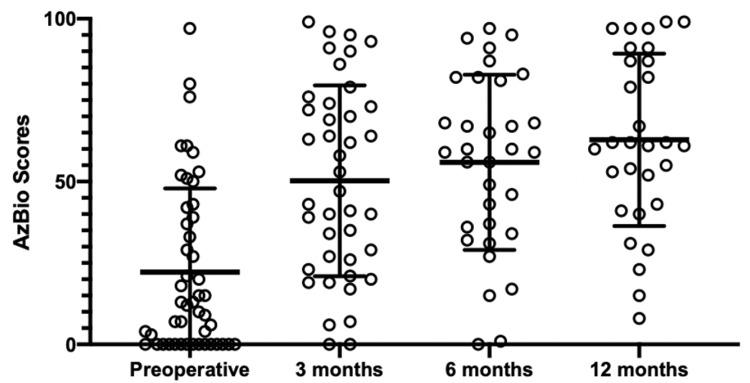
Scatter plot of AzBio scores in quiet across time points Error bars represent mean and standard deviation. Mean change from preoperative to three months was significant; however, change from three months to six months, and six months to 12 months was not significant.

All speech outcome measures were obtained preoperatively, and at three, six, and 12 months postoperatively. Speech outcome measures are summarized in Table 1. The mean preoperative CNC score in the ear to be implanted was 18%±18, while the mean postoperative CNC score at three, six, and 12 months was 35%±21, 44%±23, and 45%±25 respectively (Figure 1). Similar results were measured using AzBio scores in quiet. The mean preoperative AzBio score in the ear to be implanted was 22%±26 while the mean postoperative AzBio score at 3, 6, and 12 months was 50%±29, 56%±27, and 63%±26 respectively (Figure 2). All patients were followed to the end of the one-year period; however, not all data points were collected at every visit (Table 1). 

**Table 1:** Summary of speech outcomes CNC: consonant-nucleus-consonant

Time point	CNC words (SD)	Mean difference speech outcome scores between subsequent time points (p-value)	AzBio in quiet (SD)	Mean difference speech outcome scores between subsequent time points (p-value)
Preoperative	18% (18%)		22% (26%)	
3 months	35% (21%)	15% (P = .0046)	50% (29%)	30% (P < .001)
6 months	44% (23%)	6.7% (P > .05)	56% (27%)	4.4% (P > .05)
12 months	45% (25%)	.74% (P > .05)	63% (26%)	5.5% (P > .05)

The Wilcoxon signed-rank and paired t-tests were used to observe significant change in speech performance between time points. The greatest change in speech performance for the majority of patients was seen between pre-implantation and three months with a mean difference of 15% (P < .0046) and 30% (P <.0001) using CNC and AzBio, but there were non-significant changes between three, six, and 12 months. Binomial distribution tables with 95% CI modeled after Thornton and Raffin (1978) were also used to assess significant improvement in speech understanding using CNC and AzBio. OF the implantations, 74% (32 of 43) and 69% (22 of 32) showed significant improvement at six months or one year using an AzBio or CNC binomial distribution table, respectively. 

Mean difference speech outcomes by electrode array

The patient population was subdivided into two cohorts: (1) precurved electrode and (2) straight electrode to observe any differences in speech outcomes. All outcomes are summarized in Table 2. To adjust for differences in preoperative mean CNC (8.5% and 23%) and AzBio (11% and 31%) scores for precurved electrode and straight electrode, the mean change between preoperative and postoperative scores were compared between the two groups. The mean change between preoperative and postoperative scores at 12 months for precurved electrode and straight electrode using CNC was 20% and 26% (P > .05) and that using AzBio was 37% and 42% (P > .05), respectively. Mean difference scores demonstrate no significant difference between groups at 12 months. Mean difference scores using CNC and AzBio at all other time points were also nonsignificant.

**Table 2:** Speech outcomes by electrode array *Of the cochlear implant surgeries, 21 used precurved electrode arrays while 28 used straight electrode arrays CNC: consonant-nucleus-consonant; Preop: pre-operation

Test	Electrode Array*	Preop	3 months	Mean change from preop	Mean change p-value	6 months	Mean change from preop	Mean change p-value	12 months	Mean change from preop	Mean change p-value
CNC	Precurved electrode	8.6%	29.8%	20.2%	P > .05	47.5%	30.4%	P > .05	34.3%	19.7%	P > .05
	Straight electrode	23.1%	37.8%	12.7%		41.7%	24.3%		52.7%	25.8%	
AzBio	Precurved electrode	10.6%	44.5%	35.7%	P > .05	55%	43%	P > .05	52.6%	36.8%	P > .05
	Straight electrode	31.2%	55.1%	25.5%		56.5%	23.7%		70.6%	41.5%	

Speech outcomes by age of patients at surgery

Bivariate analyses were performed to demonstrate the relationship between preoperative age at implantation and postoperative speech performance using mean difference postoperative CNC and AzBio scores at three, six, and 12 months. The Pearson correlation coefficients are presented in Table 3. Statistically significant negative correlations were found between preoperative age and mean difference speech performance scores at one year using both CNC and AzBio. 

**Table 3:** Bivariate analysis of mean difference speech outcome scores between preop and postop by age of patients at implantation preop: pre-operation; postop: post-operation; CNC: consonant-nucleus-consonant

	Test	3 months	6 months	12 months
Correlation coefficients	CNC	-.35 (P > .05)	-.54 (P = .0054)	-.72 (P < .0001)
	AzBio	-.42 (P = .0058)	-.21 (P > .05)	-.55 (P = .0017)

Mixed-effect modeling

Mixed-effect modeling was used to assess speech outcomes in the settings of differences in gender, age at implantation, and electrode type (Table 4). When accounting for gender, age, and electrode type, results showed that the increases in CNC (17.7%; 95% CI: 16.8% - 34.6%; P < .0003) and AzBio scores (29.5%; 95% CI: 20.5% - 38.6%; P < .0001) at three months compared to pre-operation were statistically significant (Table 4). Moreover, when adjusting for gender, age at implantation, and preoperative speech outcome score, the association between electrode type and CNC score was not statistically significant (P > .05). The model also demonstrated that when adjusting for other factors, the association between speech outcomes and gender was not significant (P > .05). However, mixed-effect modeling showed a statistically significant negative association between the precurved electrode cohort and AzBio scores post implantation (-13.3%; 95% CI: -24.8% - -1.85%; P < .0238). Finally, mixed-effect modeling demonstrated that when gender, type of electrode array, and preoperative speech outcome scores were held constant, the weak negative associations between age at implantation and CNC and AzBio scores were not statistically significant. 

**Table 4:** Mixed-effect modeling data *p-values of intercept indicate that when controlling for the above factors that there was a significant improvement in both CNC and AzBio in quiet scores, comparing postoperative to preoperative scores. CNC: consonant-nucleus-consonant; preop: pre-operation; postop: post-operation

Test	Effect	Estimate	95% CI of estimate		t-value	p-value
CNC	Intercept	0.4023	0.179	0.6255	3.64	0.0008*
	Gender: Female	-0.05402	-0.1602	0.05216	-1.03	0.3103
	Gender: Male	0	.	.	.	.
	Age	-0.00248	-0.00539	0.000437	-1.72	0.0935
	Electrode type: Precurved	-0.08682	-0.1895	0.01585	-1.71	0.0952
	Electrode type: Straight	0	.	.	.	.
	Time: 3 months Postop	0.1768	0.1681	0.346	3.78	0.0003
	Time: 6 months Postop	0.2547	0.08349	0.27	5.75	< .0001>
	Time:12 months Postop	0.257	0.1664	0.3429	5.76	< .0001>
	Time: Preop	0	.	.	.	.
AzBio	Intercept	0.4817	0.2411	0.7223	4.03	0.0002*
	Gender: Female	-0.05797	-0.1761	0.06013	-0.99	0.3281
	Gender: Male	0	.	.	.	.
	Age	-0.00278	-0.00605	0.000498	-1.71	0.0945
	Electrode type: Precurved	-0.1333	-0.2481	-0.01851	-2.34	0.0238
	Electrode type: Straight	0	.	.	.	.
	Time: 3 months Postop	0.295	0.2045	0.3856	6.46	< .0001>
	Time: 6 months Postop	0.3323	0.2352	0.4294	6.79	< .0001>
	Time: 12 months Postop	0.3952	0.2948	0.4955	7.81	< .0001>
	Time: Preop	0	.	.	.	.

## Discussion

The National Institute on Deafness and Other Communication Disorders (NIDCD) estimates that approximately 15% of American adults report some trouble hearing and that nearly 25% of adults aged 65-74 years, and 50% of adults aged 74 and older have disabling hearing loss [1]. Moreover, a recent study by Gomen et al. estimates that by 2060, with a rapidly growing elderly population, over 45 million Americans will have moderate hearing loss (hearing loss between 25-40 dB) and over 73 million Americans will have moderate or greater hearing loss (hearing loss greater than 40 dB) highlighting the immense impact hearing loss will have on the US population in the future [18]. The literature has described the negative impact hearing loss can have on quality of life (QOL) and cognitive ability [19] illustrating the importance of hearing loss rehabilitation [20]. However, utilization rates remain low with only 5% of eligible adult patients utilizing cochlear implantation in the US [10]. Reasons for this underutilization may include a lack of awareness of cochlear implantation utility and eligibility criteria by primary care providers and referring physicians [9], limited access to cochlear implant centers, and patient out-of-pocket costs [10,11]. This work hopes to add to the growing body of knowledge demonstrating the benefit cochlear implants can have on speech outcomes for patients suffering from post-lingual sensorineural hearing loss. Moreover, this work demonstrates speech outcome improvement for adult patients across a variety of factors, including electrode array, age, gender, and preoperative speech outcome scores, highlighting the utilization of cochlear implants for a diverse and growing population.

Although cochlear implants provide significant benefit for speech performance for the majority of patients, significant speech outcomes variability exists in the postoperative setting. As such, speech outcome mean difference scores and binomial distribution tables were used to assess speech performance for the patient population. For most patients, speech outcome scores progressively improved over the entire postoperative time period, with the greatest change in speech performance for the majority of patients occurring between preoperative and three months with a mean difference of 15% (P < .0046) and 30% (P <.0001) using CNC and AzBio, respectively. These results highlight a possible “ceiling effect” as mentioned previously in the literature by Helms et al. [21]. These findings may inform discussions with patients about realistic expectations in the postoperative setting. 

In line with the current literature, the study found that both precurved and straight electrodes are clinically effective for cochlear implantation [22]. Both the precurved and straight electrode cohorts demonstrated varied speech outcome improvement, and mean difference scores between the two groups were not significantly different at 12 months (P > .05). Moreover, when adjusting for gender, age at implantation, and preoperative speech outcome scores, the type of electrode array had a nonsignificant effect on postoperative CNC speech outcome scores; however, a statistically significant negative association was found between the precurved electrode array and postoperative AzBio scores. Reasons for these differences are puzzling and could be due to the use of larger precurved arrays and not newer thinner precurved arrays allowing for differences in intracochlear trauma from insertion. However, CNC speech outcome results from the study support previous works suggesting that selection of the electrode should be made by the surgeon independent of preconceived notions of electrode superiority [9,22]. The present analysis demonstrated that sex did not significantly affect speech outcomes in patients, which conflicts with findings from a 2019 analysis of 55 patients by Raymond and colleagues showing higher relative postoperative improvement for female cochlear implant recipients [23]. These results suggest that future studies should assess the effect of sex on speech outcomes with a larger cohort of patients.

Although bivariate analysis demonstrated that younger patients outperformed older patients using mean difference AzBio and CNC scores, mixed-effect modeling showed that when electrode type, preoperative speech outcome scores, and gender were held constant, age at implantation did not have a statistically significant association with speech performance. Differences in outcomes may be due to neural plasticity [24]. These results highlight the benefits that patients of all ages may receive from cochlear implantation [25,26]. In addition, several papers that have focused on QOL changes post-cochlear implantation have shown that younger and older patients have similar QOL outcome scores post-implantation illustrating the positive impacts of surgery beyond speech performance improvements such as improved mood, cognitive function, and autonomy [19,27-29].

This present study is subject to several limitations. First, this study was performed retrospectively, which introduces the potential for bias. Second, implantations were performed by two different surgeons, and audiologic metrics were gathered by four audiologists introducing bias in operative procedure and speech outcome collection. Variations in programming approach, patient familiarity with the test condition (e.g. whether programming adjustments were made before or after testing), and subject characteristics (e.g. wear time, aural rehabilitation, language onset, and cognition) may have further impacted the present results, especially due to the small sample size. Streamlining data collection to uniform minimum reporting criteria will likely allow for the accumulation of larger numbers of directly comparable subjects from our cohort to those in the literature over time. Third, preoperative and postoperative speech outcome performance data and LF PTA were missing for several patients due to the evolution of testing protocol during the time period studied and new changes in reporting standards [30]. Fourth, there was a lack of racial diversity in the present population with a majority of the patients identified as White non-Hispanic. Fifth, hearing loss and other comorbidities often coexist, which can affect cognitive and rehabilitative outcomes in this group of patients. Nonetheless, the study cohort consisted of a diverse population in terms of electrode array, gender, and age at a nascent center, which may provide realistic outcomes for patients seen at small to mid-sized cochlear implant programs.

Findings from the study have important implications for cochlear implantation in adults. First, the study raises awareness for the improvement in speech outcome cochlear implants can provide for patients of all ages. Second, these findings may support early adoption of cochlear implantation due to our observation of younger patients showing larger improvements in speech understanding. Finally, continued adoption of standardized reporting criteria across multiple centers along with shared reporting of outcomes may allow for pooling of data across multiple centers and strengthening the literature behind expected improvements in speech understanding after cochlear implantation. 

## Conclusions

Despite the well-established benefit of cochlear implantation for sensorineural hearing loss, adoption of the treatment remains low in the US. Our study provides further evidence for the benefits of cochlear implants for speech outcomes in adults. Findings from this review show a positive association between speech outcome scores and time in patients who receive implantation regardless of electrode type, gender, or age. A better understanding of speech outcomes is crucial for physicians and audiologists at small to mid-sized cochlear implant programs who hope to provide realistic expectations for patients.
